# Effects of Adding Methods of Fluorane Microcapsules and Shellac Resin Microcapsules on the Preparation and Properties of Bifunctional Waterborne Coatings for Basswood

**DOI:** 10.3390/polym14183919

**Published:** 2022-09-19

**Authors:** Lin Wang, Yan Han, Xiaoxing Yan

**Affiliations:** 1 Co-Innovation Center of Efficient Processing and Utilization of Forest Resources, Nanjing Forestry University, Nanjing 210037, China; 2 College of Furnishings and Industrial Design, Nanjing Forestry University, Nanjing 210037, China

**Keywords:** microcapsule, waterborne wood coating, thermochromism, self-healing, film properties

## Abstract

In this paper, urea-formaldehyde resin microcapsules with shellac resin as core material were prepared by in-situ polymerization. Morphologies of shellac resin microcapsules were characterized by optical microscope (OM) and scanning electron microscope (SEM). Both microcapsules were spherical in shape. The encapsulation property of shellac resin was proved by Fourier transform infrared (FTIR). Shellac resin microcapsules and fluorane microcapsules were added to waterborne primer or topcoat at the same time to prepare waterborne coatings with thermochromic and self-healing dual functions. The effects of microcapsules on optical properties, mechanical properties, self-healing properties, anti-aging performance, and thermoreversible discolouration mechanism of coating films were studied. These results showed that the topcoat with 10.0% fluorane microcapsules and 5.0% shellac resin microcapsules had a better comprehensive performance. At this time, the colour of coating transformed yellow into colourless at 32 °C, and it had a good colour recovery. Shellac resin microcapsules endowed the coating with self-healing performance, and the self-healing rate was 35.9%. The research results provide a reference for the progression of multifunctional wood coatings.

## 1. Introduction

Wood plays an important role in production and life because of its light weight, high strength, beautiful appearance, low processing energy consumption, renewable nature, ability to be recycled, and natural degradation [1,2,3,4,5,6]. Among them, the hard, broad-leaved wood has higher hardness, higher density, rich colour, and texture changes and has been favoured by traditional timber structure architecture, rare furniture construction, ship engineering, and other fields since ancient times [7,8,9,10]. As a kind of wood, hard, broad-leaved wood belongs to the natural polymer heterogeneous composite material. The main component of cellulose contains a large number of hydrophilic polar groups, which makes it have significant wet expansion and dry shrinkage and low dimensional stability [11]. In addition, the cellulose and the hemicellulose are easily decomposed into sugars with a low degree of polymerization, which is very conducive to the propagation of mould [12]. Up to now, the common method to maintain the application of broad-leaved wood is to coat its surface with environmentally friendly and green waterborne coating film, which can improve the decorative effect and durability of broad-leaved wood to some extent [13,14,15,16]. However, due to the changes of environmental factors such as light and heat, the lack of toughness of waterborne coating may lead to the interface mismatch between waterborne coating and hard, broad-leaved wood, thus reducing the sealing performance, inducing failure of waterborne coating, and even leading to layered damage and matrix cracking of polymer-based composite material system [17,18,19].

Microcapsule refers to a small container with core-wall structure formed by wrapping dispersive solid, liquid, or gas with film-forming material [20]. The whole process of preparing microcapsules is called “microencapsulation”, in which the encapsulated material is the core material, and the material wrapping the core material is the wall material [21]. A microcapsule self-healing system has low requirements on the chemical structure of the matrix material and has good universality [22]. In addition, the microcapsules can be functionalized through coating different kinds of core materials [23]. The emergence and development of this intelligent technology can not only guarantee the safety of materials and equipment but also reduce the cost of testing and maintenance. Thus, it has a wide application prospect in the high-tech industry [24]. da Cunha et al. [25] prepared polyurea formaldehyde microcapsules with flaxseed oil as core material and benzoic triazole (BTA) as a corrosion inhibitor. The corrosion resistance of microcapsule epoxy resin loaded with 5wt% on carbon steel substrate was studied. These results showed that compared with pure resin coating, the presence of microcapsules provided better protection with an average RP of 3 times higher. Guo et al. [26] chose two kinds of self-repairing microcapsules to appraise the repairing performance of asphalt materials containing different doses of microcapsules under shear, tensile, and bending loading modes and determined the optimal dosage of emulsified asphalt-bonding microcapsules to be 1.0% through comprehensive tests. There have been many reports on the self-healing of metal and asphalt using microcapsule technology, but there are few studies on the self-healing performance of waterborne coatings applied to wood furniture surface.

In addition, with the progress of science and technology and the improvement of life taste, people have higher requirements on the intelligence and visual sense of decoration materials [27]. Thermochromic wood can not only satisfy people’s visual enjoyment but also be designed from multiple angles according to consumer needs, so it has a broad space for development in home decoration and other fields [28]. Wood usually needs surface treatment before use, and the discolouration features of wood mainly hinge on the material surface [29]. Microencapsulation of discolouration material is one of the effective methods to expand the application range of thermochromic wood. Therefore, adding the colour-changing microcapsule into the coating film of wood products is one of the methods for high utilization of achievements.

Low-temperature reversible thermochromic microcapsules have become a hot spot in recent years, especially organic low-temperature reversible thermochromic microcapsules, which are mainly due to their relatively significant advantages such as narrow discolouration range, bright colour, and obvious discolouration [30]. At present, the core of the most widely studied and most practical organic reversible thermochromic microcapsules is usually a three-component compound [31]. Commonly used thermochromic microcapsules are organic thermoreversible microcapsules containing a three-component system [32]. In the three-component complex system, the organic compounds that act as colorants are usually triarylmethane, fluoranes, rhodamine B, and phenothiazines [33]. Fluorane has the advantages of low discolouration temperature and high sensitivity, but there are few studies on fluorane thermochromic microcapsules in wood surface coatings.

The single-component self-healing system is simple and beneficial to practical application, and there is no need to worry about the distribution and dosage of the two components in the binary self-healing system, and there is no need to use complex technology to carry out multilayer coating of the components [34]. Shellac resin is a natural product, non-toxic, and environment-friendly, with nice film formation, fast drying and curing speed, distinctive solubility, and significant thermal properties [35]. Shellac resin coating made from shellac is also widely available for wood furniture [36].

For intelligent waterborne wood coatings, their self-healing and discolouration properties are essential in practical application. Therefore, it is of great practical significance to develop a coating that can realize self-healing at room temperature and thermochromism. Basswood stands out among many hard broad-leaved woods because of its fine texture and good machinability [37]. Shellac resin dissolved in ethanol can be used as the core material of microcapsule to repair cracks at room temperature. More to the point, the fluorane microcapsules can realize the thermoreversible discolouration between yellow and colourless. In this paper, the shellac-resin-filled urea-formaldehyde resin microcapsules were prepared taking advantage of the method of in situ polymerization. The 1,2-benzo-6-diethylaminofluorane microcapsules (hereafter referred to as fluorane microcapsules), which are widely used in the market, were selected in this work. Three factors of “fluorane microcapsules content”, “shellac resin microcapsules content”, and “fluorane microcapsules and shellac resin microcapsules”, were added into the primer or topcoat (hereafter referred to as “adding method”) were selected to design the orthogonal test. On the basis of this technology, the coating samples for independent experiment were prepared.

Different from the self-healing microcapsule-based waterborne wood coatings studied by our research group in the previous period [38,39], the previous research mainly focused on the preparation of self-healing microcapsules for different core materials, such as epoxy resin and waterborne acrylic resin. This study mainly focuses on the content of two kinds of microcapsules (fluorane microcapsules and shellac resin microcapsules) and the influence of their addition methods on the coating performance of wood products. One is that the prepared self-healing microcapsule core materials are different; the other is that this study involves two kinds of microcapsules with different functions, and the third is that the process explored is also different.

By means of optical, mechanical, liquid resistance, and reversible thermochromic properties; artificial accelerated aging, self-healing scratch test, OM, SEM, and FTIR, a better preparation scheme was obtained. The two kinds of microcapsules were added to the waterborne topcoat at the same time, which simplified the process in the production and facilitated the operation, establishing a base for the application of thermochromic and self-healing bifunctional waterborne coatings on the basswood surface.

## 2. Materials and Methods

### 2.1. Experimental Materials

The particular experiment materials are shown in Table 1. The main components of fluorane microcapsules are melamine formaldehyde resin (as wall material, CAS No. 9003-08-01), 1,2-benzo-6-diethylaminofluorane (Red DCF, dye, C_28_H_23_NO_3_, M_W_: 421.49 g/mol, CAS No. 26628-47-7), methyl palmitate (as solvent, C_17_H_34_O_2_, M_W_: 270.45 g/mol, CAS No. 112-39-0), ethyl stearate (as solvent, C_20_H_40_O_2_, M_W_: 312.5304 g/mol, CAS No. 111-61-5), and styrene maleic anhydride monomethyl-maleate polymer (as emulsifier, C_17_H_16_O_7_, M_W_: 332.305 g/mol, CAS No. 31959-78-1). Primer and topcoat are mainly made up of waterborne acrylic acid copolymer dispersion, matting agent, additive and water, solid content of about 30.0%, provided by Keyuan Industrial Co., Ltd., Shanghai, China.

### 2.2. Preparation of Shellac Resin Microcapsules

**Figure 1:**
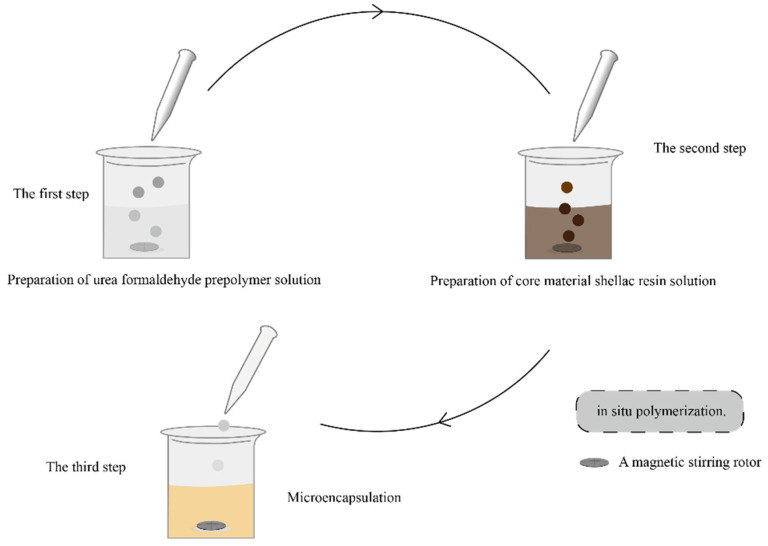
The prepared process of shellac resin microcapsules by in situ polymerization.

**Figure 2:**
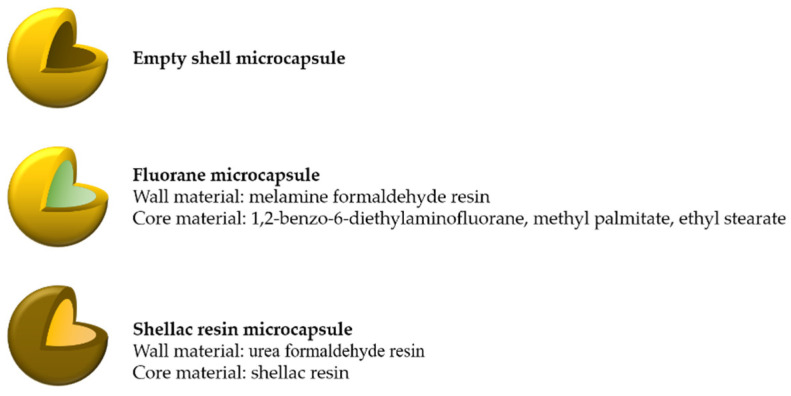
A schematic drawing showing the round microcapsules with core and surface shells.

The prepared process of shellac resin microcapsules is shown in Figure 1. The schematic drawing of Figure 2 shows two kinds of round microcapsules with core and surface shell used in the test, clearly showing the material of the outer shell and the material of the inner core. Preparation of urea formaldehyde prepolymer solution: 20.0 g urea and 27.0 g 37.0% formaldehyde solution were stirred evenly. The pH was adjusted through gradually adding triethanolamine to 8.0–9.0. A magnetic stirring rotor was added into the system for magnetic stirring. It was stirred constantly for 1 h in a water bath at 70 °C. The prepared emulsion was the wall material solution, and it needed to cool naturally to room temperature to work with.

Preparation of core material shellac resin solution: The 112.5 g anhydrous ethanol and 22.5 g shellac resin were added to a beaker and stirred until dissolved. The 1.760 g sodium dodecyl benzene sulfonate white powder was added to 174.24 mL distilled water and stirred well to obtain 1.0% sodium dodecyl benzene sulfonate emulsifier solution. The emulsifier solution was added to the dissolved shellac solution, and the rotor was added. The uniform core material emulsion was acquired by stirring continuously at 1200 rpm for 30 min.

Microencapsulation: At a speed of 300 rpm, the cooled wall material prepolymer was dropped into the core material emulsion, and then, citric acid monohydrate was added slowly. The pH value was adjusted to 2.5–3.0, and the reaction time was 2 h. After the reaction, the product was stored for 7 d and next was repeatedly washed and filtered with deionized water and anhydrous ethanol. In the end, the product was dried at 80 °C for 4 h, and the earth-yellow powders were obtained as microcapsules.

### 2.3. Preparation of Thermochromic and Self-Healing Dual Function Coatings

Three factors and two levels orthogonal test were designed, as shown in Table 2. According to the literature [40] and preliminary experiments, when 15.0% fluorane microcapsules were added to waterborne primer or waterborne topcoat, the thermochromic property of the waterborne coating film on basswood surface was better. Therefore, the level range of “fluorane microcapsule content” was selected around 15.0%, that is, 10.0–20.0%, to determine the optimal value. At the same time, the content of shellac resin microcapsule was determined according to the literature [41]: the coating film had better comprehensive performance at the 10.0% microcapsule content. Therefore, the level range of “shellac resin microcapsule content” in this study was selected around 10.0%, that is, 5.0–15.0%. The component part of waterborne coatings doped with microcapsules are demonstrated in Table 3. Samples 1–4 # are the corresponding materials for the orthogonal test. Samples 5–11 # were used for independent optimization tests on the basis of orthogonal tests. Samples 12 #, 13 #, and 5 # were used as blank control. Take sample 1 # as an example: 0.2 g fluorane microcapsules, 0.1 g shellac resin microcapsules, and 1.7 g primer were mixed uniformly and coated on the basswood substrate with SZQ tetrahedral preparer (Chengdu Zhitong Trading Co., Ltd., Chengdu, China); the sample was moved to an oven at 35 °C, heated for 20 min until the surface was dry, then taken out, and naturally cooled. The surface was lightly polished with 800 # sandpaper and wiped off the floating powder with a dry cloth. This process was repeated twice to complete the primer. Then, 2.0 g topcoat was weighed and mixed evenly, and the same SZQ tetrahedral preparer was used to coat the basswood substrate that had been coated with primer. The sample was transferred to an oven at 35 °C and heated for 20 min once the surface was dry. Then, it was taken out and naturally cooled. The surface was gently polished with 800 # sandpaper, and the float powder was wiped off with dry cloth next. This operation was repeated twice, and sample 1 # was obtained after the topcoat was applied. Other samples were prepared by the same method as sample 1 #. The dry film thickness was about 60 μm.

### 2.4. Test and Characterization

The indoor temperature in Nanjing in winter is 16 °C, and the highest indoor temperature in summer is 40 °C, and the test temperature range was set. The experiment temperature range is 16–40 °C. The HHP1 heating plate (Shanghai Hengyue Medical Device Co., Ltd., Shanghai, China) was used to heat the sample. At the same time, the surface temperature change of the film was evaluated by a hand-held infrared thermometer (Jiangsu Taizhou Tertan Automation Technology Co., Ltd., Taizhou, China). SEGT-J portable chromaticity meter (Shenyang Guotai Precision Testing Instrument Co., Ltd., Shenyang, China) was used to test the colourimetric parameters of the film during the heating process of 16–40 °C and the cooling process of 40–16 °C. The colour difference formula of the coating was based on the CIELAB colour space. The *L* represents lightness, a larger value indicates that the surface chroma of the coating film is brighter, and a smaller value indicates that the surface chroma is darker. The *a* means that the chroma transforms red into green, a positive value means that the chroma is red, and a negative value means that the chroma is green. The *b* means that the chromaticity transforms yellow into blue, a positive value means that the surface chromaticity is yellow, and a negative value means blue. *c* is colour saturation. *H* is for hue. The larger the *L* value, the brighter the film colour. The larger the *a* value, the redder the film colour. The greater the *b* value, the yellower the film colour. The colourimetric parameters of a thermochromic sample at 16 °C were used as reference points to record the changes of the colourimetric parameters at different test temperatures during the two independent tests of heating and cooling. In conformity with Hunter colour difference Equation (1), the variation trend of colour difference Δ*E* of samples at different temperatures was calculated.

(1) $$\Delta E=\sqrt{\left[{\left(\Delta L\right)}^{2}+{\left(\Delta a\right)}^{2}+{\left(\Delta b\right)}^{2}\right]}$$

where Δ*L* (lightness difference) = *L* − *L’*; Δ*a* (red–green difference) = *a* − *a’*; Δ*b* (yellow-blue difference) = *b* – *b*’. *L*, *a*, and *b* represent the chroma value of the film at 16 °C. *L’*, a’, and *b’* represent the chromaticity value of the film at other temperatures. The gloss of the film was tested through the HG268 intelligent gloss meter (Shenzhen Sanenshi Technology Co., Ltd., Shenzhen, China).

Hardness of the coating film is evaluated by portable pencil method hardness tester (Guangzhou Biada Precision Instrument Co., Ltd., Guangzhou, China) and 6H-6B pencil. During the hardness experiment, the angle between the pencil and the film was 45°, and the pencil was scratched at a load of 1.0 kg. Pencil hardness indicates the hardness of the coating. QFH-HG600 Bageknife film grader (Tianjin Shengnuo Instrument Technology Co., Ltd., Tianjin, China) is selected to evaluate the film adhesion. Adhesion of the film is the best when the adhesion grade is 0. QCJ-50 film impact resistance tester (Hebei Yaoyang Instrument Equipment Co., Ltd., Cangzhou, China) was selected to evaluate the impact resistance of the film. The weight was fixed to the desired height with a control screw, and the height was read with the aid of the positioning mark. The controller screw was pressed so that the connecting hammer fell and was applied to the sample beforehand placed on the pad. Subsequently, the sample surface coating was observed using a magnifying glass after the hammer body was lifted, and the experimental sample was taken out. Impact strength of the film is expressed by the maximum height at which a 1.0 kg hammer can impact the film without causing damage. The unit of impact strength is kg·cm. Elongation at break of the film was evaluated by AG-IC100KN precision electronic universal testing machine (Shimadsu Corporation, Kyoto, Japan). The film stretching speed was 0.12 mm/min. The paint film was fixed on the vertical surface of the upper and lower fixtures with the fixture. The distance *l_1_* was tested between the upper and lower fixtures. The machine was started, and the machine was stopped when the paint film breaks. At this point, the clamping distance *l_2_* was read, and the elongation at break (*e*) was calculated according to Equation (2).

(2) $$e=\frac{l_{2}-l_{1}}{l_{1}}\times 100\%$$

The NaCl solution, medical ethanol, detergent, and red ink were used to evaluate the liquid resistance of coating film. The filter paper was put into the test solution and soaked for 30 s. Then, it was taken out with tweezers and quickly put in the test area. The flowing liquid was wiped off, and the sample was covered with a toughened glass cover at once. The cover and paper were withdrawn after 24 h. The residual liquid on the coating film surface was blotted with absorbent paper, and sample stood for 30 min to check the damage of the sample test area, such as imprint and discolouration. The colour difference and gloss of each test area were measured to determine the influence of different reagents on the film performance.

DHG-9643BS-III oven (Shanghai Xinyao Medical Equipment Manufacturing Co., Ltd., Shanghai, China) 120 °C, 160 °C, and UV light-resistance climate test chamber (Nanjing Environmental Testing Equipment Co., Ltd., Nanjing, China) performed the artificial accelerated aging experiment, which was conducted to evaluate the stability of the film. Colour difference and gloss of the film were measured periodically every 8 h after the film was put into the oven. The total test time was 40 h. The coating was tested every 40 h in the UV light-resistance climate test chamber, and the total test time was 200 h. The colour difference and surface morphology of the film before and after aging were observed and compared.

After the scratches of waterborne coating film on the glass plate were cut with the blade and immediately placed under AxioScopeA1 optical microscope (Zeiss, Jena, Germany), the crack width *W_1_* of the wound gap was observed on a computer connected to the optical microscope (OM), and the size was marked and a photo taken. Then, after standing at room temperature for 5 d, the width of the wound gap *W_2_* was observed again under an optical microscope, and the size was marked. In the later period, the crack widths of the coating film before and after the scratches were compared to characterize whether the microcapsules have a certain self-healing function for the waterborne coating film. According to Equation (3), the self-healing rate of the crack was calculated (η).

(3) $$\eta =\frac{W_{2}-W_{1}}{W_{1}}\times 100\%$$

The 1.0 g microcapsules (*M_1_*) were sufficiently ground in the mortar and put into the sand core funnel. A certain amount of anhydric ethanol was doped into the microcapsules, which were soaked for 72 h sufficiently, and the anhydric ethanol was replaced every 24 h. Then, they were rinsed and filtered with deionized water, and the residual wall material was weighed (*M_2_*) after drying. The coating rate (*C*) was calculated by Equation (4).

(4) $$C=\frac{M_{1}-M_{2}}{M_{1}}\times 100\%$$

The microstructures of the microcapsules and coatings were observed using an OM and Quanta 200 scanning electron microscope (FEI, Hillsboro, OR, USA). Suspension droplets of microcapsules were absorbed by a pipette on a slide, and the morphology of microcapsules was observed under the OM. In addition, the basswood board was sliced and observed under OM. The dry microcapsule powder was attached to the sample table with the conductive adhesive and sprayed with gold for SEM observation. Then, the size was marked, and the microcapsules in the image were selected for statistics. In light of the percentage of the number of microcapsules and the total number of microcapsules in a certain particle size range, the particle size distribution of microcapsules was obtained.

The chemical composition of microcapsules and coatings was analysed by VERTEX 80v Fourier Infrared spectrometer (German Bruck Co., Ltd., Karlsruhe, Germany). The test range was 4000–500 cm^−1^, the sample scanning specimen was 16 s, and the resolution was 4 cm^−1^. All experiments were repeated 4 times with an error of less than 5.0%.

## 3. Results and Discussion

### 3.1. Morphology and Properties Analysis of Microcapsules

**Figure 3:**
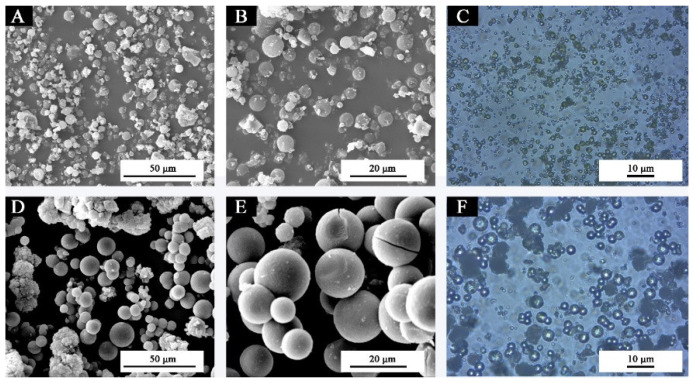
Morphologies of fluorane microcapsules (**A**–**C**) and shellac resin microcapsules (**D**–**F**). Reprinted with permission from [43].

**Figure 4:**
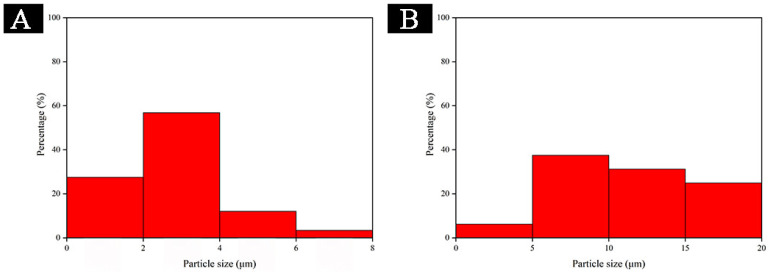
Particle size distributions of (**A**) fluorane microcapsules (**B**) shellac resin microcapsules.

According to Formula (4) for calculating the coating rate, the coating rate of shellac resin microcapsule was 12%, and the yield was 25.5 g. SEM and OM pictures of fluorane microcapsules and the prepared shellac resin microcapsules are shown in Figure 3. Figure 3 shows the morphologies of fluorane microcapsules and shellac resin microcapsules, respectively. The shellac resin microcapsules are round and spherical. In conformity with Figure 3B,E, the particle size of microcapsules was recorded. The distributions are displayed in Figure 4. The two kinds of the microcapsules have uniform particle size and narrow distribution, with the highest proportion of fluorane microcapsules in 2–4 µm and shellac microcapsules in 5–10 µm, which are ideal microcapsules. The particle size of fluorane microcapsules is smaller than that of shellac microcapsules, but all of them are in the smaller micron range, which is conducive to the orderly arrangement of microcapsules and the reflection of light. In accordance with the principle that light propagates in a medium with unequal diffraction rates, diffraction rings are generated at the junction of the medium [42]. As shown in OM Figure 3C,F [43], the optical diffraction rings appeared in fluorane microcapsules and shellac resin microcapsules, indicating the existence of two different media. The dark outer ring represents the wall material, and the transparent inner ring represents the core material. The microcapsules are nearly round and present as the clear, core-wall structures. Both kinds of microcapsules are the formations of a single core and single wall.

**Figure 5:**
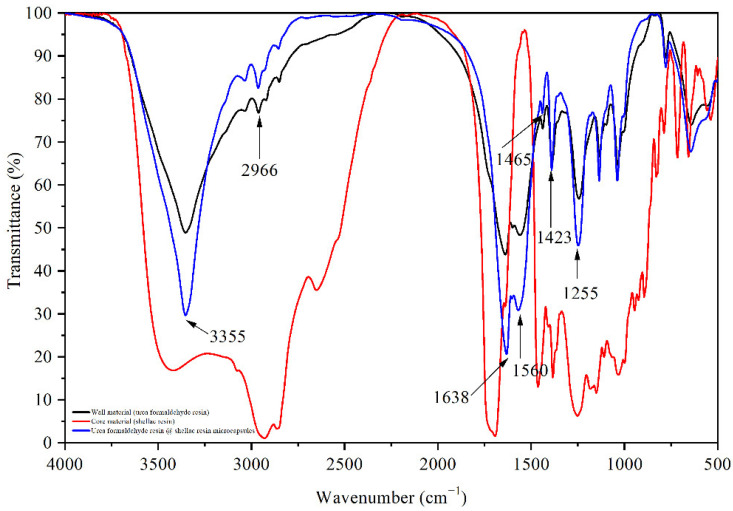
FTIR of urea formaldehyde resin, shellac resin, and shellac resin microcapsules.

Figure 5 shows the infrared spectra of shellac resin, urea-formaldehyde resin, and shellac resin microcapsules. According to the infrared spectrum of the microcapsules, there are obvious absorptions around 3355 cm^−1^, 2966 cm^−1^, 1638 cm^−1^, and 1560 cm^−1^, which are the stretching vibration of N–H, C–H, C=O, and C–N, respectively. Through the above four kinds of adsorption, the existence of urea-formaldehyde resin as wall material was proven [44]. Shellac resins are connected by ester bonds and contain hydroxyl and carboxyl groups [45]. The characteristic peak of shellac appeared around 1465 cm^−1^, 1423 cm^−1^, and 1255 cm^−1^ [35,46]. Because the shellac is dissolved into liquid in ethanol, the uncoated shellac resin is removed during the extraction and filtration process of microencapsulation, and there is no mixing of shellac monomers in the microencapsulation powder. Shellac resin is present in the microcapsule, and the chemical structure is not damaged. Therefore, the shellac resin (core material) is well-coated by urea-formaldehyde resin (wall material).

### 3.2. Orthogonal Test Analysis

**Figure 6:**
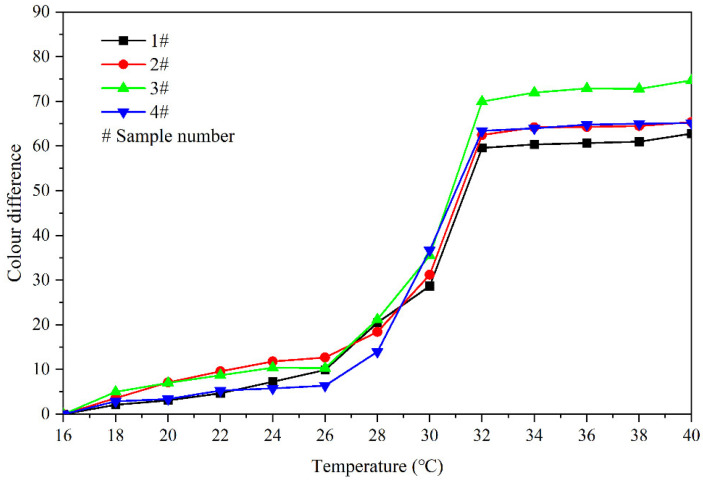
Effect of temperature increase (16–40 °C) on colour difference of film for samples 1 #–4 *#*.

Figure 6 shows the influence of heating (16 °C to 40 °C) on the colour difference of the coating film containing fluorane microcapsules. The colour difference of the coating film gradually increases with the increase of the temperature of orthogonal samples 1–4 #. On the whole, the colour difference of orthogonal sample 1 # is smaller than that of orthogonal samples 2–4 #, and the colour difference of 3 # is the largest. When the temperature increases from 30 °C to 32 °C, the colour difference of the coating film increases greatly and tends to a maximum at 32 °C. It can be shown that the thermochromic temperature range of samples 1–4 # is 30–32 °C, and the thermoreversible colour-changing phenomenon occurs at 32 °C.

In order to make the film have the best thermochromism performance, the influence of these factors on its colour difference performance is mainly explored, so the colour difference of 16–32 °C was put into the orthogonal analysis of Table 4 for analysis. The orthogonal test and range results show that “fluorane microcapsule content” is the primary factor influencing the colour difference of the coating film, followed by “adding method” and “shellac resin microcapsule content”. According to k1 and k2, it can be further found that “shellac resin microcapsule content” is 5.0%, and the addition method is “fluorane microcapsules and shellac resin microcapsules were added to the topcoat at the same time”, which has a greater influence on the coating film colour difference at 16–32 °C. Therefore, in the next orthogonal test optimization, shellac resin microcapsule content was fixed at 5.0%. The effect of fluorane microcapsule content (5.0%, 10.0%, 15.0%, 20.0%, 25.0%, and 30.0%) on various properties of coatings was studied.

### 3.3. Single-Factor Test Results and Analysis of Fluorane Microcapsule Content

**Figure 7:**
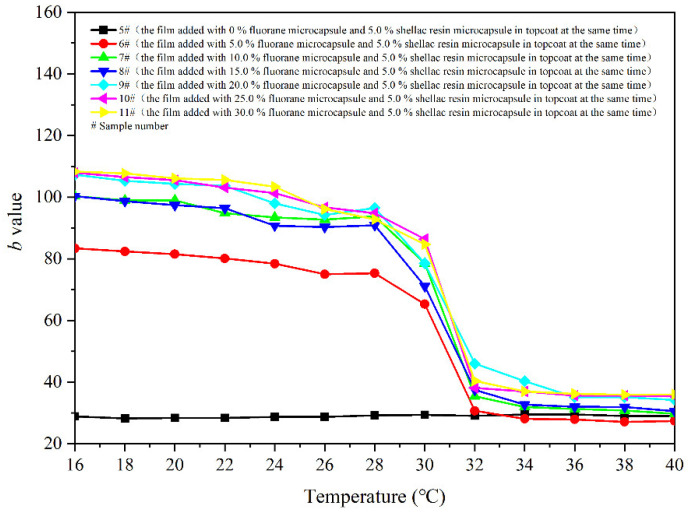
Effect of temperature increase (16–40 °C) on the *b* value of film.

Figure 7 shows the variation trend of the *b* value of the coating with the increase of temperature. The larger the *b* value, the yellower the colour of the film. As can be seen from Figure 7, in the general trend, with the increase of the test temperature, the decreasing *b* value of samples 5–11 # also indicates that the colour of the film gradually transforms yellow into colourless. The *b* value of the coating film does not change greatly in the range of 16–28 °C, and the *b* value begins to show a downward trend at 30 °C. When the temperature continues to rise to 32 °C, the *b* value changes remarkably and stabilizes in the range of 32–40 °C. Thus, it can be preliminarily determined that the colour of coating film changes at the node of 32 °C.

**Figure 8:**
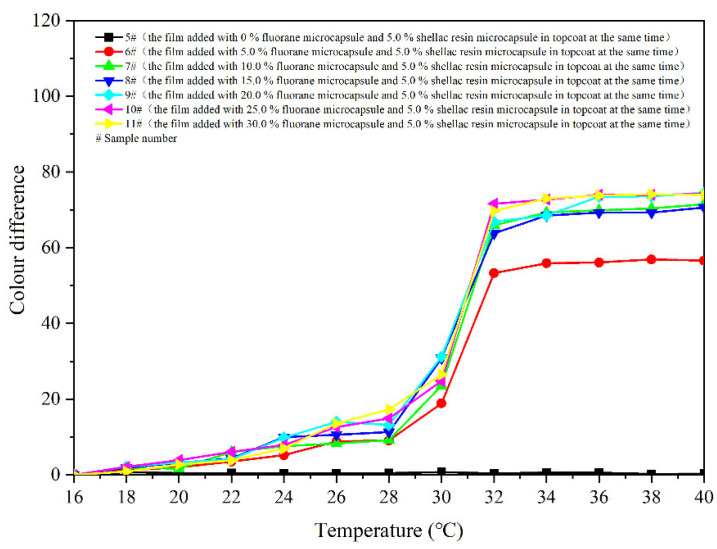
Effect of temperature increase (16–40 °C) on colour difference of film for samples 5 #–11 *#*.

**Figure 9:**
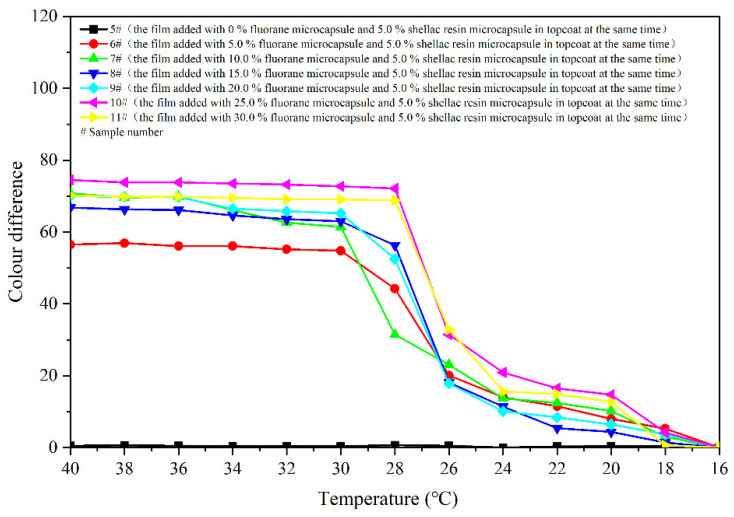
Effect of temperature drop (40–16 °C) on colour difference of film.

**Figure 10:**
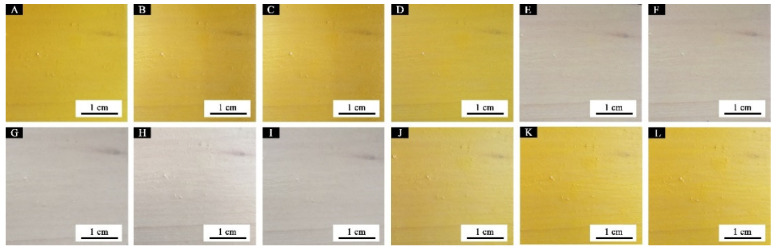
Photographs of colour trend of 7 # (film of 10.0% fluorane microcapsules and 5.0% shellac microcapsule added in topcoat at the same time) changes with temperature: temperature increases to (**A**) 16 °C, (**B**) 26 °C, (**C**) 28 °C, (**D**) 30 °C, (**E**) 32 °C, and (**F**) 40 °C; temperature drops to (**G**) 40 °C, (**H**) 32 °C, (**I**) 30 °C, (**J**) 28 °C, (**K**) 26 °C, and (**L**) 16 °C.

Figure 8 shows colour differences of the coatings 5–11 # when they are heated from 16 °C to 40 °C. Colour differences of the coating films added with 5.0–30.0% microcapsules are basically below 20.0 at 16–28 °C, and the discolouration effects are not apparent. The colour difference increases significantly as the temperature up to 30 °C. And then, as the temperature rises to 32 °C, the colour differences basically reach the maximum, indicating that the coatings have a thermochromism effect at this temperature. Colour differences of the coating film without fluorane microcapsule are between 0 and 1.0, which do not have the effect of discolouration. In addition, the colour differences of coatings with fluorane microcapsule content of 5.0% are smaller than that of coatings with other contents. Thermochromic effect of the coatings with fluorane microcapsule content of 10.0–30.0% is better. According to the trend of colour difference during heating and cooling (Figure 8 and Figure 9), the prepared coating produces a reversible thermochromic effect. As the temperature rises to 32 °C, colour difference of the film 7 # reaches 65.9. The colour trends of the films are the same. Therefore, film 7 # was chosen as an illustration, and the colour trend of film 7 # with the temperature’s rise and fall is shown in Figure 10.

The addition of fluorane microcapsules with different contents in the topcoat led to a downward trend in the gloss of the coating surface in Table 5. The gloss of 5 # was the highest, which was 18.7%. This may be because as the fluorane microcapsule content in the face paint increased within limits, the coating was uneven, leading to light scattering to some extent and thus abating the coating gloss [47].

Table 6 shows the effects of fluorane microcapsule content on coating mechanical properties. As the content of fluorane microcapsule increased, the impact resistance increased from 12.0 kg·cm to 21.0 kg·cm, and the hardness increased from 3 H to 5 H. The results demonstrate that the microcapsules with the film have a good compatibility, and the microcapsules are evenly distributed in the coating matrix and have a good impact resistance. As long as the film is impacted, the impact force is applied to the wall material of the microcapsules and rapidly transferred to the wall material edge. The wall materials partly have toughness and compressive strength, which can act on a buffering role and lessen the internal stress of the substrate material, thus improving the impact resistance of the coating film to some extent [48]. In addition, the experimental results show that, at 0–30.0% fluorane microcapsules content, the adhesion grade of the film is 0, and the adhesion performance is superb, indicating that the excellent adhesion of the original film can be held through heightening the fluorane microcapsule content in the topcoat. The trend of elongation at break is arched, and the best elongation at break is 31.1% at the addition of 10.0% fluorane microcapsules. This is because the wall materials of the two kinds of microcapsules (shellac resin microcapsule and fluorane microcapsule) in the topcoat, the urea-formaldehyde resin and melamine formaldehyde resin, play a toughening role in the stretching process. More important is that the core material (shellac resin) is released by an external force to ease the crack, so adding a certain amount of microcapsule can enhance the toughness of the coating by microcapsules, and elongation at break also increases. However, with the increase in addition amount, the microcapsules form an agglomeration in the coating, thus reducing the flexibility of the coating film [49].

The anti-pollution performance of wood surface coating is studied by measuring its resistance to cold liquid. The liquid resistance of coatings with different contents of fluorane microcapsules to NaCl, detergent, ethanol, and red ink was tested. The experiment was carried out at room temperature of 16 °C in winter, and the colourimetric parameters of the film were appraised before and 24 h after the test. The colour difference and 60° gloss of film before and after liquid resistance are exposed in Table 7 and Table 8, respectively. After four kinds of cold liquid testing, the gloss of the coating film has no significant change compared with that before the liquid resistance; overall, the gloss change range is not large. Thus, the results of the liquid resistance of the coating from the Table 9 are clear. A large colour difference indicates that the liquid resistance of the film is poor, and the liquid resistance grade is also high. After the cold liquid-resistance solution test, the colour value of red ink changes most obviously. The coating with microcapsule content of 0–30.0% has a liquid-resistance grade of 1 to NaCl, ethanol, and detergent without damage. In the wake of the microcapsule content heightening, the liquid resistance of the coating film decreases, and the liquid-resistance grade decreases from grade 2 to grade 3, with slight imprint. These results show that the red ink has a greater effect on the film than the other three liquids. This may be because the film itself has the good curing degree and film-forming performance, so the pure coating has better fluid resistance [50]. However, the coating was less resistant to red ink, which added with fluorane microcapsules because the film at room temperature was yellow; after the red ink experiment, by capillary action, the colour of the coating changes from yellow to red. Therefore, the colour difference becomes larger, leaving a more obvious impression.

**Figure 11:**
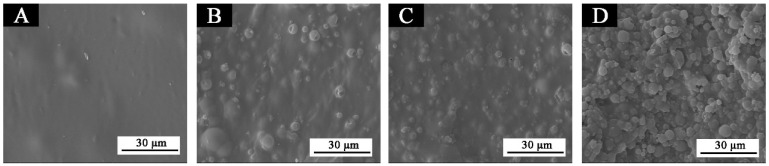
SEM of coatings with different contents of fluorane microcapsules: (**A**) 5 #, (**B**) 6 #, (**C**) 7 #, and (**D**) 11 #.

Figure 11 shows the waterborne coating films with different contents of fluorane microcapsules doped in the topcoat. With the continuous addition of fluorane microcapsule content in the topcoat, the morphology of particles on the film surface tends to be obvious. When the content is 30.0%, the surface of the coting is uneven, which may be caused by the aggregation of microcapsules in the film due to too much added content.

**Figure 12:**
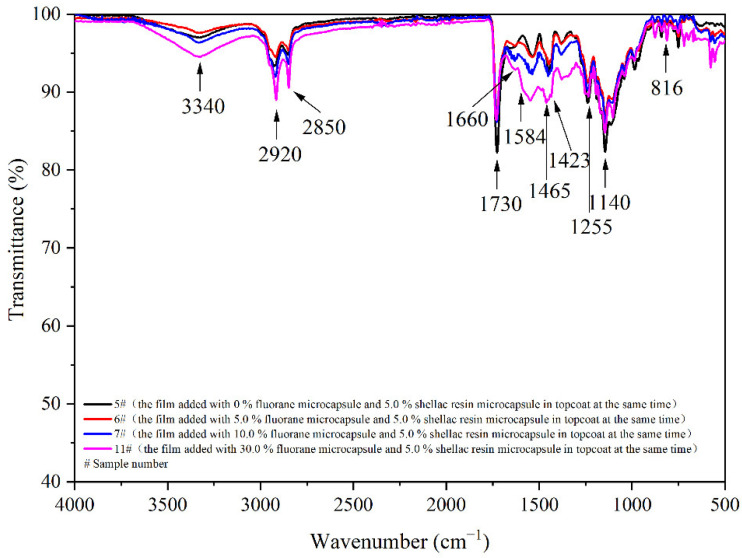
FTIR of film dopped with different contents of fluorane microcapsules.

Figure 12 shows the infrared spectra of 5 #, 6 #, 7 #, and 11 # coatings. The tensile vibration absorption of –NH and –OH is superimposed at 3340 cm^−1^. The 2920 cm^−1^ is the tensile vibration of –CH_3_. The 1140 cm^−1^ is the stretching vibration absorption of C–O–C. The stretching vibration absorption peak and bending vibration absorption peak of triazine ring are located at 1584 cm^−1^ and 816 cm^−1^, respectively. The 1660 cm^−1^ is attributed to the stretching vibration of C=O in urea-formaldehyde resin. The 1730 cm^−1^ with strong characteristic carbonyl absorption represents the characteristic peak of C=O in one of the core materials of fluorane microcapsules (1,2-benzo-6-diethylaminofluorane). At 1465 cm^−1^ and 1423 cm^−1^, the carboxylic acid carbonyl anion COO– antisymmetric and symmetric stretching vibration are shown, and at 1255 cm^−1^, the C=O–C stretching vibration in the ester molecule is shown. The infrared spectra of 6 #, 7 #, and 11 # showed characteristic peaks at 1584 cm^−1^, 816 cm^−1^, and 1730 cm^−1^ for fluorane microcapsules and 1465 cm^−1^, 1423 cm^−1^, and 1255 cm^−1^ for shellac resin microcapsules. When different content of fluorane microcapsules and 5.0% shellac resin microcapsules are added to the topcoat at the same time, no peaks disappear or appear, manifesting that there is no difference in the coating composition of fluorane microcapsules with different content. This indicates that there is no chemical reaction between the two microcapsules and coating on basswood.

The performance analysis of fluorane microcapsule content single-factor test shows that when shellac microcapsule content is 5.0%, the adding method is added fluorane microcapsule and shellac resin microcapsule in topcoat at the same time, and fluorane microcapsule content is 10.0%, the comprehensive property of the coating film is better, that is, in sample 7 #. Subsequently, the blank samples of 5 #, 12 #, and 13 # were tested for comprehensive performance and compared with the best sample of 7 #.

### 3.4. Analysis of Artificial Accelerated Aging Test

**Figure 13:**
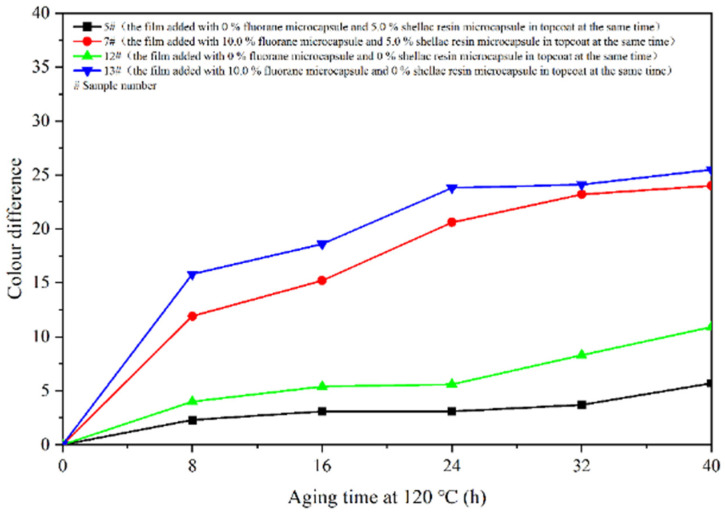
Effect of aging time on colour difference of film at 120 °C in oven.

**Figure 14:**
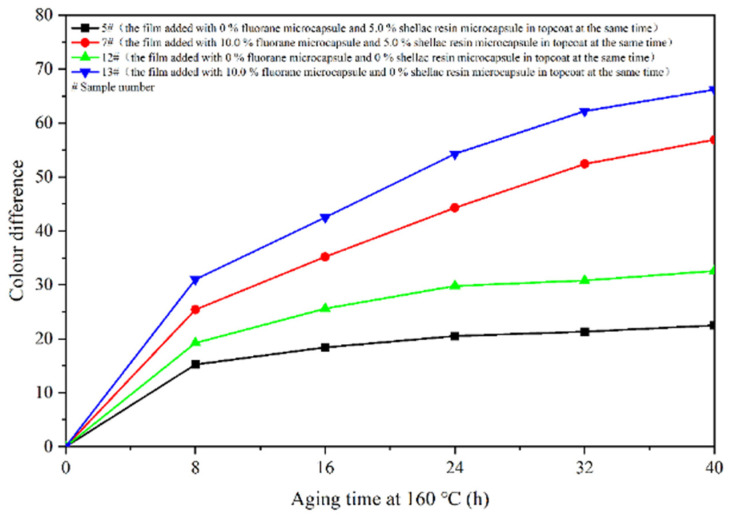
Effect of aging time on colour difference of film at 160 °C in oven.

**Figure 15:**
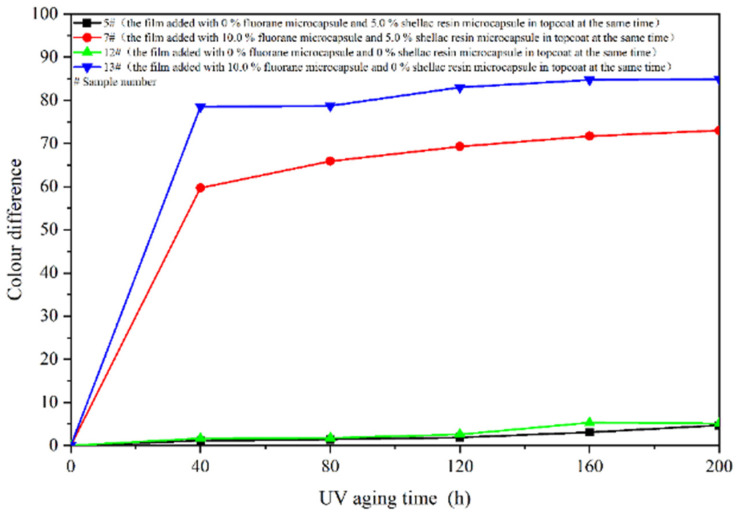
Effect of UV aging time on colour difference of coating.

The aging resistance and self-healing test of the coating films 5 #, 7 #, 12 #, and 13 # were carried out under three environments, namely, 120 °C in oven, at 160 °C in oven, and in the UV light-resistance climate test box. The surface of the coating film was observed. Figure 13, Figure 14 and Figure 15 show the trend of colour difference of coating film with aging time. When the coating was heated in the oven at 120 °C, the colour difference of the blank sample 5 # increased to 5.7 with the increase of heating time, sample 7 # increased to 24.0, the colour difference of blank sample 12 # increased to 10.9, and the blank sample 13 # increased to 25.5. When the film was heated in the oven at 160 °C, the blank sample 5 # increased to 22.5, sample 7 # increased to 56.9, the blank sample 12 # increased to 32.6, and the blank sample 13 # increased to 66.2. When the film was in the UV-resistance chamber, the colour difference of the blank sample 5 # increased to 4.7, sample 7 # increased to 73, blank sample 12 # increased to 5.1, and blank sample 13 # increased to 84.9.

The results show that colour difference of the same coating film increases with the aging time. After aging of different coating films, only fluorane microcapsules were added, and the colour difference of the aging samples was larger, indicating that fluorane microcapsules were unstable in the aging process and could not maintain the chromaticity value of the samples. Furthermore, the fluorane was damaged after 160 °C and UV aging, resulting in a high degree of degradation [51]. The colour difference of waterborne film changes greatly in 160 °C aging environments, and the colour difference changes little in the ultraviolet aging environment, which indicates that waterborne film is not heat-resistant with aging. After adding shellac resin microcapsules and fluorane microcapsules, the aging colour difference of coating film has a tendency to slow down. This is because the coating may produce microcracks in the aging process, and the shellac resin microcapsule core flows out of the repair agent to repair the microcracks and restrain the surface damage. On the other hand, the increase of colour difference may be due to the colour change of wood in the aging environment, which makes the colour difference of the coating film greater [52].

**Figure 16:**
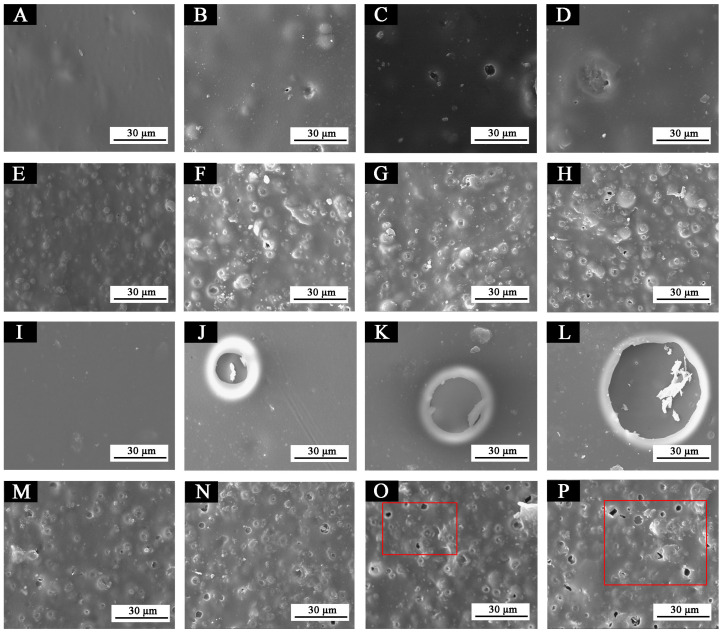
SEM of films before and after aging in different environments: (**A**) 5 #, (**B**) 5 #—120 °C, (**C**) 5 #—160 °C, (**D**) 5 #—UV, (**E**) 7 #, (**F**) 7 #—120 °C, (**G**) 7 #—160 °C, (**H**) 7 #—UV, (**I**) 12 #, (**J**) 12 #—120 °C, (**K**) 12 #—160 °C, (**L**) 12 #—UV, (**M**) 13 #, (**N**) 13 #—120 °C, (**O**) 13 #—160 °C, and (**P**) 13 #—UV. Reprinted with permission from [43].

Figure 16 shows SEM images of the coatings 5 #, 7 #, 12 #, and 13 # before and after aging in different aging environments (120 °C, 160 °C, UV light-resistance climate test chamber). Figure 16B–D shows that micro-bubbles begin to appear in the coating film 5 # after aging at 120 °C. After aging at 160 °C, the number of bubbles slightly increases, and the diameter of bubbles slightly increases but not obviously. After aging in the UV light-resistance climate test chamber, the coating film is in good condition. Figure 16F–H shows that small bubbles appear on the surface of the coating 7 # after aging at 120 °C and 160 °C, but it is not obvious. No obvious bubbles or cracking symptoms appear after the UV aging environment, and the coating is in good condition. Figure 16J–L [43] shows that large bubbles appear in the coating film 12 # after the aging environment of 120 °C. After the aging environment of 160 °C and UV light-resistance test chamber, the bubble diameter increases sharply, indicating that the coating film has been destroyed very significantly. Figure 16N–P [43] shows that the coating 13 # began to show tiny bubbles after the aging environment of 120 °C, and the number of bubbles increased after the 160 °C and UV aging environment. The results manifest that the coating films added with fluorane microcapsule or shellac resin microcapsule coated on basswood have a certain effect on suppressing cracks, and the coatings with shellac resin microcapsule have a better effect on suppressing cracks. This could be because no matter what kind of microcapsule, they are almost globular in shape and can be better uniformly distributed in the coating and bind to the coating and form a relatively dense, protective film. Therefore, it can block part of the damage caused by high temperature and ultraviolet rays in time and can better adapt to the invasion of environmental factors. On the other hand, the coating added with shellac resin microcapsules may rupture when aging. The shellac resin, the core material repair agent, was released, resulting in cross-linking curing, thus inhibiting the large-scale damage of the coating microstructure [41,53].

**Figure 17:**
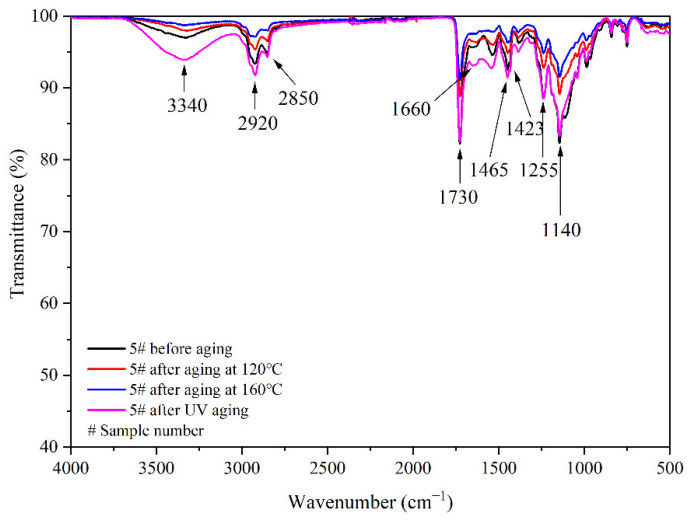
FTIR of 5 # coating before and after aging.

**Figure 18:**
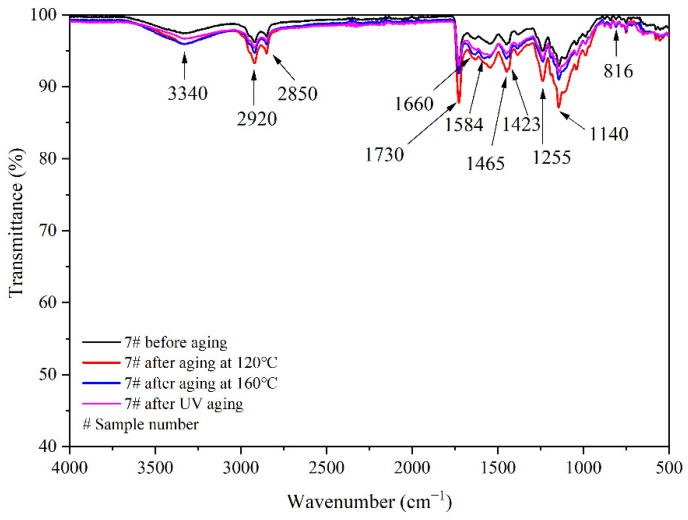
FTIR of 7 # coating before and after aging.

**Figure 19:**
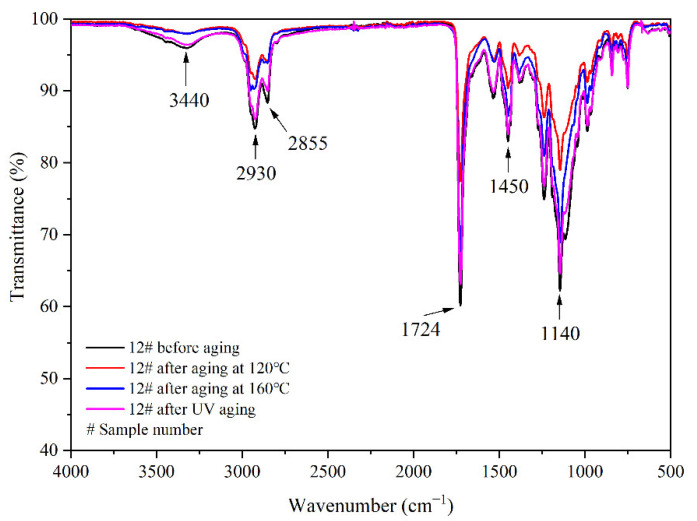
FTIR of 12 # coating before and after aging.

**Figure 20:**
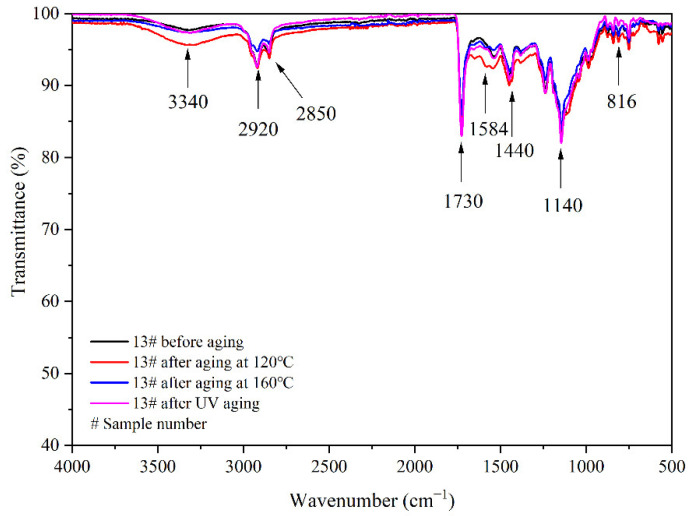
FTIR of 13 # coating before and after aging.

Figure 17, Figure 18, Figure 19 and Figure 20, respectively, show the FTIR of samples 5 #, 7 #, 12 #, and 13 # before and after three kinds of aging environment. The peaks of the same sample did not disappear or appear before and after aging, manifesting that there is no difference in the composition of the film before and after aging. This result shows that the film composition was totally unaffected by the aging environment, suggesting that the aging environment did not cause the coating to react chemically.

### 3.5. Self-Healing Test

**Figure 21:**
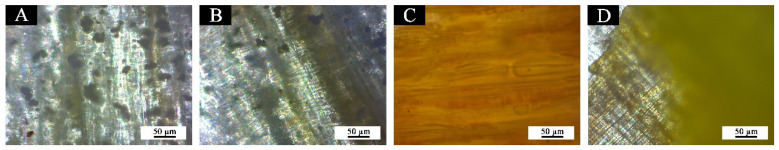
OM diagram of waterborne film on basswood: (**A**) 5 #, (**B**) 7 #, (**C**) 12 #, and (**D**) 13 #.

In order to explore the composition relationship between fluorane microcapsules and shellac resin microcapsules, waterborne coating film, and basswood, different samples were sliced and observed under the OM. Figure 21A–D shows the microstructures of sliced samples 5 #, 7 #, 12 #, and 13 # and observed that the arrangement of the pits on the basswood duct wall seen in the four figures is parallel [54]. Figure 21D shows that fluorane microcapsules disperse well on the surface of basswood in waterborne coating without agglomeration, which may be due to its small particle size, mainly distributed in 2–4 μm. In Figure 21A,B, it can be seen that some shellac resin microcapsules form agglomeration in the coating film, with obvious particles.

**Figure 22:**
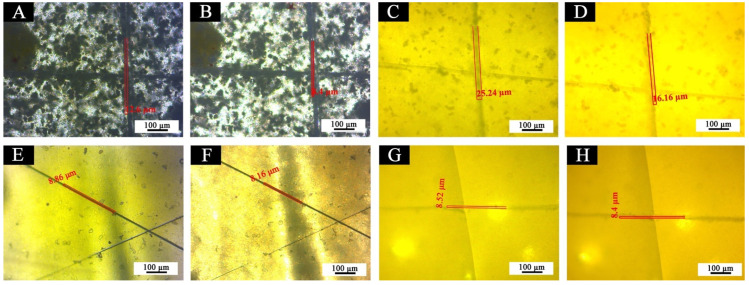
OM diagram of coating film: before self-healing (**A**) 5 #, (**C**) 7 #, (**E**) 12 #, and (**G**) 13 #; after self-healing (**B**) 5 #, (**D**) 7 #, (**F**) 12 #, and (**H**) 13 #.

Figure 22 is the OM diagram of the crack width comparison of different coatings before and after repair. Figure 22A is the OM view of coating 5 # immediately observed after being scraped by the blade, and Figure 22B is the crack width after standing for 5 d. Figure 22C,D is coating 7 #, Figure 22E,F is coating 12 #, and Figure 22G,H is coating 13 #, according to the crack width before and after the coating repair to characterize whether the coating has the self-healing function. Table 10 shows the comparison of scratch crack width of film before and after self-healing.

After 5 d, the cracks of the coating film with shellac resin microcapsule shrank in Figure 22A,B and Figure 22C,D. At room temperature, the crack width of 7 # was 25.24 μm before repair, and the crack width was 16.16 μm after 5 d. The crack width of the coating was obviously reduced by 9.08 μm. At the same, the crack width of 5 # was 12.6 μm before repair, and the crack width was 8.4 μm after 5 d. The crack width decreased by 4.2 μm. However, for the coating films without shellac resin microcapsules, i.e., in Figure 22E,F and Figure 22G,H, the crack width of the coating films was between 0–0.7 μm after being placed for 5 d, and it hardly narrowed. In conclusion, both the 7 # “topcoat with 10.0% fluorane microcapsules and 5.0% shellac resin microcapsules” and the 5 # “topcoat with 0% fluorane microcapsules and 5.0% shellac resin microcapsules” can achieve a certain degree of self-healing. The coating self-healing mechanism is that when microcracks occur in the coating film with shellac resin microcapsules, the wall material of the microcapsule is broken, and the shellac resin of the core material is released, resulting in cross-linking and curing so as to repair the microcracks in time [41].

### 3.6. Thermoreversible Discolouration Mechanism

**Figure 23:**
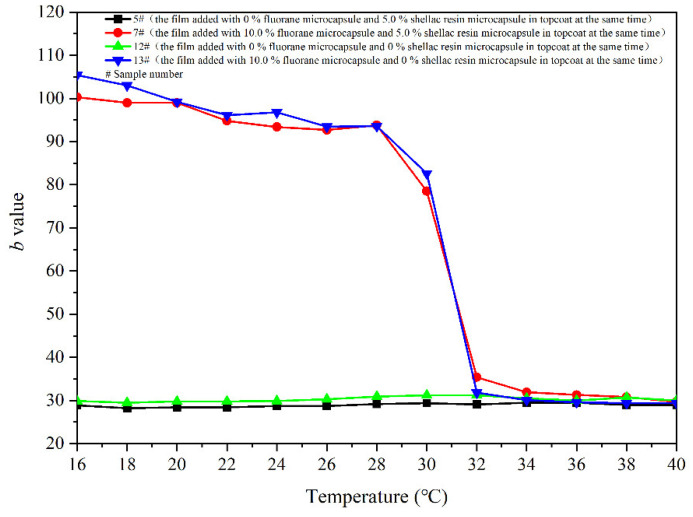
Effect of temperature increase (16–40 °C) on the *b* value of coating.

Figure 23 shows the trend of the *b* value of the coating with increasing temperature. The *b* value of the coating films 7 # and 13 # added with fluorane microcapsules decreases with the increase of temperature in Figure 23. The decreasing *b* value of samples 7 # and 13 # also indicates that the colour of the coating film gradually transforms from yellow into colourless. The *b* value of the coating film does not change evidently in the range of 16–28 °C and starts to decrease at 30 °C. When the temperature continues to rise to 32 °C, the *b* value changes apparently and tends to be stable in the range of 32–40 °C. It can be preliminarily determined that the coating at 32 °C transformed yellow into colourless. However, the *b* value of the coating film 5 # and 12 # without fluorane microcapsules does not decrease with the increase of temperature and basically stays at about 28.0–31.0, which preliminarily indicates that the colour of films 5 # and 12 # does not change.

**Figure 24:**
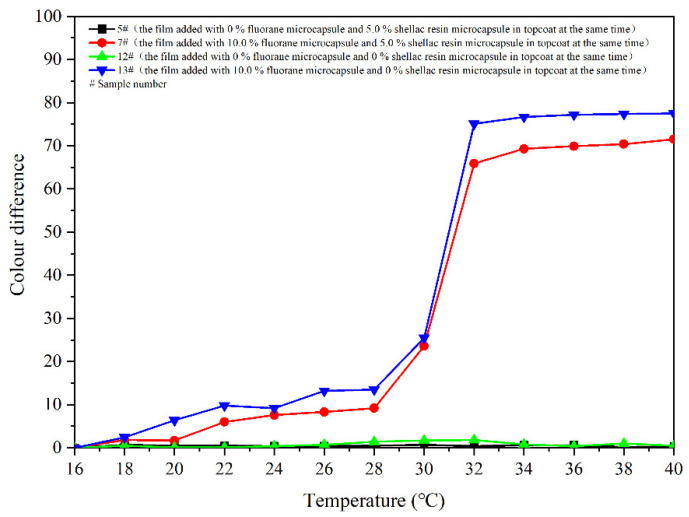
Effect of temperature increase (16–40 °C) on colour difference of coating.

**Figure 25:**
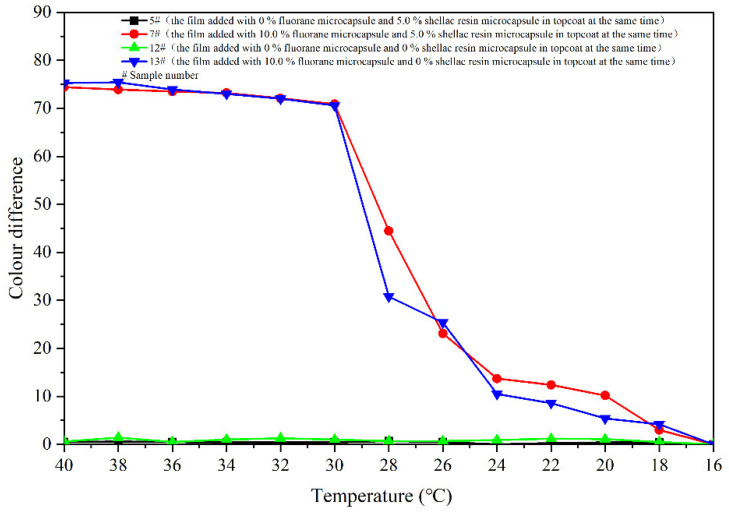
Effect of temperature drop (40–16 °C) on colour difference of coating.

Figure 24 displays the colour differences of the films heated from 16 °C to 40 °C. The colour differences of coating film 7 # and 13 # at 16–28 °C are basically below 20.0, and the thermochromic effects are not remarkable. As the temperature rises up to 30 °C, the colour differences rise significantly. When the temperature continues to rises to 32 °C, the colour differences are basically in a high range, indicating that the films have thermochromism at this temperature. The colour differences of 5 # and 12 # without fluorane microcapsules are between 0 and 1.0, which do not have the colour-change effect. In accordance with the tendency of colour difference during heating and cooling (Figure 24 and Figure 25), the reversible thermochromic effects of the films 7 # and 13 # are produced.

Table 11 illustrates the colour difference of the best sample 7 # and other blank control groups during heating (16–32 °C), revealing their thermochromic effect. The colour difference of the coating films with fluorane microcapsule is very large, which is more than 60. However, the colour difference of the coating without fluorane microcapsule had little change. As the ambient temperature changes, the waterborne coating on the surface of basswood with fluorane microcapsules changes colour reversibly.

**Figure 26:**
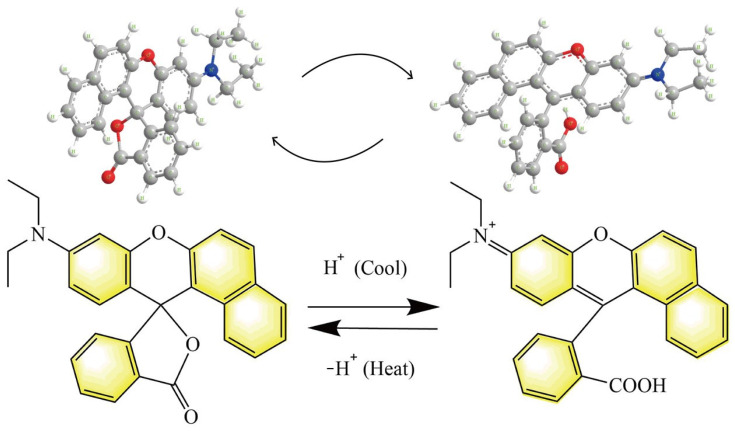
Thermoreversible discolouration mechanism of fluorane microcapsules.

Figure 26 conjectures the thermoreversible discolouration mechanism of fluorane microcapsules. The 1,2-benzo-6-diethylaminofluorane is an electron donor dye with lactone ring structure. The solid-liquid state of solvent in fluorane microcapsule is controlled by temperature. At temperatures below the melting point of the solvent, 1,2-benzo-6-diethylaminofluorane reacts with acid chromogenic agents, which can bring about the transfer of electron. The lactone ring of 1,2-benzo-6-diethylaminofluorane is opened to form a conjugated chromogenic structure, which makes the microcapsule system yellow. The 1,2-benzo-6-diethylaminofluorane is separated from the chromogenic reagent as the temperature of the system increases because the solvent turns to liquid at this moment. Therefore, the quinone structure is transformed into lactone ring structure in the liquid state and does not appear in colour [55]. The aforesaid procedure is reversible, which gives a thermoreversible discolouration function to the waterborne coating on basswood.

## 4. Conclusions

The effects of fluorane microcapsules and shellac resin microcapsules on the performances of waterborne coatings on basswood surface was studied. The orthogonal test and range results showed that “fluorane microcapsule content” was the primary factor influencing the colour difference of the coating film, followed by “adding method” and “shellac resin microcapsule content”. The results of independent tests showed that the topcoat added with 10.0% fluorane microcapsules and 5.0% shellac resin microcapsules was better in comprehensive performance. The coating is yellow at 16–30 °C and colourless at 32–40 °C. The colour of the coating could quickly respond to the change whether the temperature increased or decreased. The discolouration temperature of the film is 32 °C, and the discolouration temperature range is 30–32 °C. When the temperature drops, the film can also restore its original yellow. At this point, the colour difference is 65.9 at 16–32 °C, the gloss is 5.3% at 60°, the hardness is 4 H, the adhesion is grade 0, the impact resistance is 18.0 kg·cm, the elongation at break is 31.1%, and the resistance to NaCl, ethanol, and detergent is grade 1; there is no damage, the resistance to red ink is grade 2, and there is slight discontinuous impression. Shellac resin microcapsule can alleviate the speed of colour difference increase before and after coating aging. The waterborne coating with fluorane microcapsule or shellac resin microcapsule on basswood has a certain effect on suppressing cracks, and the coating with shellac resin microcapsule has a better effect on suppressing cracks. No peaks disappeared or appeared before and after aging, and no chemical reaction occurred in the aging environment. The results of the self-healing scratch test showed that compared with the pure coating, the crack width of the coating with shellac resin microcapsules was reduced by 9.08 μm after 5 d, and the self-healing rate was 35.9%. This paper provides scientific reference for the self-healing and thermochromic bifunctional waterborne wood coatings.

## Figures and Tables

**Table 1:** List of the experimental material.

Material	Molecular Formula	M_W_ (g/mol)	CAS	Content (%)	Specification Description	Producer
Fluorane microcapsules	-	-	-	-	-	Oriental Colour Technology Co., Ltd., Shenzhen, China
Shellac resin	-	-	-	-	Yunnan special grade two	Inan Dahui Chemical Technology Co., Ltd., Shandong, China
Formaldehyde solution	CH_2_O	30.03	50-00-0	37.0	-	Nanjing Chemical Reagent Co., Ltd., Nanjing, China
Urea	CH_4_N_2_O	60.06	57-13-6	99.9	-	
Triethanolamine	C_6_H_15_NO_3_	149.19	102-71-6	99.9	-	
Ethyl acetate	C_4_H_8_O_2_	88.11	141-78-6	99.5	-	
Sodium dodecyl benzene sulfonate	C_18_H_29_NaO_3_S	348.48	25155-30-0	99.9	-	Tianjin Beichen Founder reagent Factory, Tianjin, China
Citric acid monohydrate	C_6_H_10_O_8_	210.14	5949-29-1	99.9	-	Tianjin Beilian Fine Chemicals Development Co., Ltd., Tianjin, China
Anhydrous ethanol	C_2_H_6_O	46.07	64-17-5	99.5	-	Hangzhou Outuo Pu Biotechnology Co., Ltd., Hangzhou, China
NaCl solution	-	-	-	15.0	-	Langfang NABO Chemical Technology Co., Ltd., Langfang, China
Medical ethanol	-	-	-	70.0	-	Qingdao Haishi yinnuowei Disinfection Technology Co., Ltd., Qingdao, China
Red ink	-	-	-	-	-	Shanghai Fine Stationery Co., Ltd., Shanghai, China
White cat detergent	-	-	-	-	Containing 25% fatty alcohol ethylene oxide and 75% water	Shanghai Hehuang White Cat Co., Ltd., Shanghai, China
Dulux Muyun Jingwei anti-scratch wood varnish (primer/topcoat)	-	-	-	-	-	Keyuan Industrial Co., Ltd., Shanghai, China
Basswood	-	-	-	-	100 mm × 65 mm × 4 mm (format size), uniform colour, after sanding pre-treatment	The operation laboratory

**Table 2:** Orthogonal experiment design of thermochromic and self-healing film.

Sample Number (#)	Fluorane Microcapsule Content (%)	Shellac Resin Microcapsule Content (%)	Adding Method
1	10.0	5.0	Fluorane microcapsules and shellac microcapsules were added to the primer at the same time
2	10.0	15.0	Fluorane microcapsules and shellac resin microcapsules were added to the topcoat at the same time
3	20.0	5.0	Fluorane microcapsules and shellac resin microcapsules were added to the topcoat at the same time
4	20.0	15.0	Fluorane microcapsules and shellac microcapsules were added to the primer at the same time

**Table 3:** Ingredient of thermochromic and self-healing dual-function waterborne coating.

Sample Number (#)	Fluorane Microcapsule Content (%)	Shellac Resin Microcapsule Content (%)	Fluorane Microcapsule (g)	Shellac Resin Microcapsule (g)	Primer (g)	Topcoat (g)	Thermochromic and Self-Healing Dual-Function Waterborne Coatings (g)
1	10.0	5.0	0.2	0.1	1.7	2.0	4.0
2	10.0	15.0	0.2	0.3	2.0	1.5	4.0
3	20.0	5.0	0.4	0.1	2.0	1.5	4.0
4	20.0	15.0	0.4	0.3	1.3	2.0	4.0
5	0	5.0	0	0.1	2.0	1.9	4.0
6	5.0	5.0	0.1	0.1	2.0	1.8	4.0
7	10.0	5.0	0.2	0.1	2.0	1.7	4.0
8	15.0	5.0	0.3	0.1	2.0	1.6	4.0
9	20.0	5.0	0.4	0.1	2.0	1.5	4.0
10	25.0	5.0	0.5	0.1	2.0	1.4	4.0
11	30.0	5.0	0.6	0.1	2.0	1.3	4.0
12	0	0	0	0	2.0	2.0	4.0
13	10.0	0	0.2	0	1.8	2.0	4.0

**Table 4:** Orthogonal experimental analysis.

Sample Number (#)	Fluorane Microcapsule Content (%)	Shellac Resin Microcapsule Content (%)	Addition Method	Results of Colour Difference between 16–32 °C during Heating
1	10.0	5.0	Fluorane microcapsules and shellac microcapsules were added to the primer at the same time	59.6
2	10.0	15.0	Fluorane microcapsules and shellac resin microcapsules were added to the topcoat at the same time	62.5
3	20.0	5.0	Fluorane microcapsules and shellac resin microcapsules were added to the topcoat at the same time	70.0
4	20.0	15.0	Fluorane microcapsules and shellac microcapsules were added to the primer at the same time	63.4
k1	61.050	64.800	61.500	
k2	66.700	62.950	66.250	
Range (R)	5.650	1.850	4.750	

**Table 5:** Effect of fluorane microcapsule content on gloss of film.

Sample Number (#)	Fluorane Microcapsule Content (%)	20° Gloss (%)	60° Gloss (%)	85° Gloss (%)
5	0	5.1	18.7	14.6
6	5.0	2.2	6.4	4.2
7	10.0	2.1	5.3	5.3
8	15.0	1.5	3.2	4.0
9	20.0	1.7	3.5	9.1
10	25.0	1.7	2.0	1.4
11	30.0	1.8	2.1	1.1

**Table 6:** Effect of fluorane microcapsule content on mechanical properties.

Sample Number (#)	Fluorane Microcapsule Content (%)	Hardness	Adhesion (Grade)	Impact Resistance (kg∙cm)	Elongation at Break (%)
5	0	3H	0	12	17.628
6	5.0	4H	0	16	21.812
7	10.0	4H	0	18	31.100
8	15.0	4H	0	19	8.346
9	20.0	5H	0	20	6.262
10	25.0	5H	0	21	5.504
11	30.0	5H	0	21	2.478

**Table 7:** Effect of fluorane microcapsule content on colour difference of film before and after liquid resistance.

Sample Number (#)	Fluorane Microcapsule Content (%)	Red Ink	NaCl	Ethanol	Detergent
5	0	30.9	2.1	0.8	3.0
6	5.0	47.6	2.9	5.0	3.8
7	10.0	55.4	6.4	10.1	7.1
8	15.0	63.2	4.3	6.4	5.1
9	20.0	72.7	13.0	8.9	9.3
10	25.0	71.8	7.1	5.1	5.1
11	30.0	70.8	6.0	7.9	6.8

**Table 8:** Effect of fluorane microcapsule content on 60° gloss of film after liquid resistance.

Sample Number (#)	Fluorane Microcapsule Content (%)	Red Ink	NaCl	Ethanol	Detergent
5	0	15.2	15.4	16.0	16.2
6	5.0	6.0	6.0	5.9	5.9
7	10.0	5.2	4.9	5.3	5.1
8	15.0	2.8	3.4	3.3	3.3
9	20.0	3.9	3.8	3.3	3.7
10	25.0	1.4	2.0	2.0	2.1
11	30.0	1.2	1.9	2.0	2.1

**Table 9:** Effect of fluorane microcapsule content on liquid resistance.

Sample Number (#)	Fluorane Microcapsule Content (%)	Red Ink	NaCl	Ethanol	Detergent
5	0	2	1	1	1
6	5.0	2	1	1	1
7	10.0	2	1	1	1
8	15.0	3	1	1	1
9	20.0	3	1	1	1
10	25.0	3	1	1	1
11	30.0	3	1	1	1

**Table 10:** Comparison of crack width of scratch before and after self-healing.

Sample Number (#)	Crack Width of Scratch before Self-Healing (μm)	Crack Width of Scratch after Self-Healing (μm)	Crack Width of Scratch before and after Self-Healing (μm)	Self-Healing Rate (%)
5	12.60	8.40	4.20	33.3
7	25.24	16.16	9.08	35.9
12	8.86	8.16	0.70	7.9
13	8.52	8.40	0.12	1.4

**Table 11:** Comparison of coating thermochromic effect.

Sample Number (#)	Colour Difference of during the 16–32 °C Heated Process
5	0.4
7	65.9
12	1.8
13	75.1

## Data Availability

The data presented in this study are available on request from the corresponding author.
